# Microbial Dysbiosis in Photodermatoses: Formation, Pathogenesis and Intervention Strategies

**DOI:** 10.3390/cimb48050493

**Published:** 2026-05-09

**Authors:** Lanhai Zhong, Tian Wang, Lu Tang, Jiande Han, Qun Zhao, Naiyu Lin

**Affiliations:** 1 Department of Dermatology, The First Affiliated Hospital, Sun Yat-sen University, Guangzhou 510080, China; zhonglh7@mail2.sysu.edu.cn (L.Z.); wangt586@mail.sysu.edu.cn (T.W.); tanglu7@alumni.sysu.edu.cn (L.T.); hanjd@mail.sysu.edu.cn (J.H.); 2 Department of Trauma and Reconstructive Surgery, University Hospital of the Martin Luther University Halle, 06120 Halle (Saale), Germany; 3 Department of Trauma and Reconstructive Surgery, BG Hospital Bergmannstrost Halle, 06112 Halle (Saale), Germany

**Keywords:** photodermatoses, ultraviolet radiation, skin microbiome, *Staphylococcus aureus*, tissue-resident memory T cells

## Abstract

Recent studies have reported skin microbiome dysbiosis in patients with photodermatoses, featuring enriched *Staphylococcus aureus* colonization and decreased microbiome diversity. We propose that ultraviolet radiation (UVR), along with atypical antimicrobial peptides, may exert selective pressure on the skin microbiome, while cytokine dysregulation and a reduction in commensal bacteria amplify microbial dysbiosis. Dysbiotic microorganisms further release pathogen-associated patterns and virulence factors, and activate tissue-resident memory T cells, which collectively contribute to local inflammation. These mechanisms establish the skin microbiome as a potential target for early intervention. Potential therapeutic strategies may include antibiotics, phototherapy, bleach baths, phage therapy, and microbiota-based therapies. This review integrates current findings from microbial ecology, molecular biology, and host immunology to outline a conceptual framework linking UVR exposure, microbiome alterations, and cutaneous immune responses, while emphasizing the current limitations and evidence gaps in this field.

## 1. Introduction

Photodermatoses are inflammatory skin diseases that are caused or exacerbated predominantly by ultraviolet radiation (UVR), including polymorphic light eruption (PLE), chronic actinic dermatitis (CAD), actinic prurigo and solar urticaria [1]. Photoaggravated dermatoses such as lupus erythematosus are also classified within this category. In this review, actinic keratosis (AK) and cutaneous squamous cell carcinoma (cSCC) are also included in the discussion of photodermatoses, given their etiological link to chronic UVR exposure.

Photodermatoses affect a large proportion of the population. For example, PLE has a prevalence of approximately 18% in Europe [1]. In addition, photodermatoses significantly impair patients’ life quality and psychological health. Around one-third of patients report a Dermatology Life Quality Index (DLQI) score greater than 10, indicating a “very large” impact on quality of life. Moreover, levels of anxiety and depression in individuals with photodermatoses are approximately twice those reported in the British general population [1,2]. Collectively, these findings indicate that photodermatoses place a substantial burden on the medical system.

Advances in microbiology have revealed the close associations between the skin microbiome and skin diseases [3,4,5]. The skin microbiome helps to maintain the stability of the skin microenvironment by supporting the skin barrier and regulating immune responses [6], while dysbiosis of the microbiome may promote inflammation and contribute to skin diseases [7,8].

**Figure 1:**
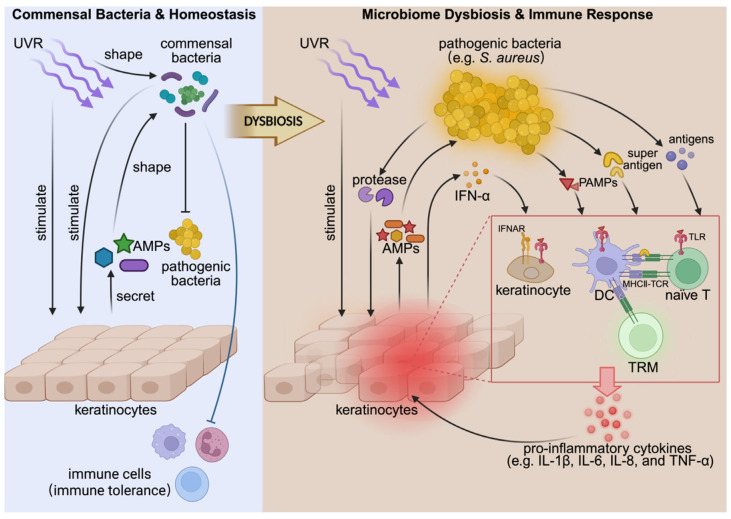
The formation and pathogenic mechanism of microbial dysbiosis in photodermatoses (AMP, antimicrobial peptide; PAMP, pathogen-associated molecular pattern; TRM, tissue-resident memory T cell; PRR, pattern recognition receptor). Created in BioRender. Su, F. (2026) https://BioRender.com/it4bejz (accessed on 4 May 2026).

Recent research reveals a dysbiotic microbiome in several photodermatoses, which manifests as decreased microbiome diversity and increased colonization by *Staphylococcus aureus* [9,10,11,12]. This review integrates current findings from microbial ecology, molecular biology, and host immunology (Figure 1) to outline a conceptual framework linking UVR exposure, microbiome alterations, and cutaneous immune responses. Based on the available evidence, we propose a model in which UVR-induced microbial dysbiosis may interact with pathogenic tissue-resident memory T (TRM) cell responses, thereby contributing to the chronicity of photodermatoses. Along this axis, restoring microbial homeostasis may represent an important strategy for mitigating persistent immune activation [13,14,15].

Nevertheless, the current evidence linking microbiome dysbiosis to photodermatoses is largely based on cross-sectional and observational studies. Therefore, these findings should be interpreted as associations rather than evidence of causality. Disease-associated changes in the skin microenvironment, including inflammation, barrier impairment, altered lipid availability, pH changes, and antimicrobial peptide expression, may also reshape microbial colonization and community structure. In addition, the skin microbiome varies substantially across anatomical sites and between individuals [16], which may be comparable to or greater than disease-associated differences. Therefore, whether microbiome alterations precede disease onset or instead arise as a consequence of disease-related skin changes remains incompletely resolved. Interventional evidence showing that microbiome manipulation can alter skin disease or cutaneous immune responses is still limited [13,17,18].

## 2. Microbial Dysbiosis in Photodermatoses

### 2.1. Polymorphous Light Eruption

PLE patients exhibit reduced microbial diversity at baseline, characterized by a depletion of key commensal bacteria such as *Staphylococcus hominis* (*S. hominis*), *Staphylococcus epidermidis* (*S. epidermidis*), *Cutibacterium acnes* (*C. acnes*), and the *Corynebacterium* genus. Conversely, pathogenic bacteria like *Staphylococcus aureus* (*S. aureus*) are relatively more abundant. Following UVR exposure, patients’ skin microbial diversity and commensal bacteria abundance further decrease, accompanied by an enrichment of *S. aureus* [11]. The relative abundance of *S. aureus* is positively correlated with the severity of the disease [11]. Moreover, recent studies in PLE have shown that reducing the skin microbiome burden can attenuate UV-induced inflammatory responses, providing supportive evidence that microbial components may contribute to disease initiation and early progression [11,13], consistent with observations reported in patients with atopic dermatitis (AD) [19].

### 2.2. Chronic Actinic Dermatitis

Sequencing of the skin microbiome in chronic actinic dermatitis (CAD) patients also reveals increased colonization by *Staphylococcus* and decreased microbial diversity, which is positively correlated with disease severity [12]. The same study also reported a decrease in *Cutibacterium* abundance in CAD patients [12], while *Cutibacterium*—especially *C. acnes*—contributes to skin homeostasis [20]. UVR exposure itself can alter the composition of the skin microbiome and modulate local immune responses [21,22], while the expansion of *Staphylococcus*—especially *S. aureus*—has been shown to promote cutaneous inflammation [7]. However, the UVR sensitivity of CAD patients is not associated with *Staphylococcus* abundance [12]. Moreover, whether *S. aureus* contributes to CAD inflammation through mechanisms similar to those observed in AD is unknown. These uncertainties indicate that the underlying mechanism needs further investigation.

### 2.3. Actinic Keratosis and Squamous Cell Carcinoma

Microbial sequencing in patients with actinic keratosis (AK) and cutaneous squamous cell carcinoma (cSCC) reveals a decreased relative abundance of *Cutibacterium* spp. and *Malassezia* spp. in lesional skin, whereas *S. aureus* is increased compared with non-lesional skin. Notably, abundance-based correlation network analysis indicates that *Staphylococcus* is generally negatively correlated with *Cutibacterium* spp. and *Malassezia* spp., suggesting potential competitive exclusion between *Staphylococcus* clusters and those of *Cutibacterium* spp. and *Malassezia* spp. [9]. *S. aureus* is enriched in AK and cSCC lesions, but the current evidence supports an association and biological plausibility rather than a confirmed causal role in the development of AK and its progression to SCC [15].

### 2.4. Systemic Lupus Erythematosus

Lupus erythematosus, including cutaneous lupus erythematosus (CLE) and systemic lupus erythematosus (SLE), is classified as a photoaggravated dermatosis. Healthy skin microbiota commonly include *Cutibacterium*, *Corynebacterium*, *Staphylococcus*, *Streptococcus*, and *Malassezia*, with relative abundance varying by anatomical site [23]. In patients with SLE, 16S rRNA sequencing of both lesional and non-lesional skin has revealed dysbiosis, characterized by decreased α-diversity, increased heterogeneity and increased colonization by *S. aureus* [24,25,26]. Mechanistic evidence suggests that chronic IFN exposure may promote *S. aureus* colonization by disrupting epidermal barrier function and upregulating adhesion-related molecules in keratinocytes [10]. Notably, *S. aureus* colonization has been shown to promote SLE-like inflammation in murine models [27], although whether a similar mechanism exists in human SLE patients requires further investigation.

Several photosensitive and photo-related skin diseases have been reported to be associated with microbiome dysbiosis (Table 1). However, these studies mainly describe associations between microbial alterations and disease phenotypes, rather than establishing causality. Most available studies are cross-sectional in nature, and the temporal relationship between microbial changes and disease activity has not been fully established. Two non-mutually exclusive hypotheses may explain these associations. One possibility is that microbial dysbiosis contributes to the pathogenesis or exacerbation of photodermatoses, as discussed in the following sections of this review. The alternate hypothesis is that skin diseases alter skin components that may reshape the microbiome; cause disease-associated changes including inflammation, barrier disruption, altered lipid availability, pH changes, and antimicrobial peptide expression; and also reshape microbial colonization and community structure. Although some evidence from AD, PLE, and SLE provides support for the former hypothesis [7,13,24,27], longitudinal and interventional studies are still needed to determine whether microbiome alterations precede disease onset or progression.

Among these observed microbial alterations, *Staphylococcus*, particularly *S. aureus*, appears to be frequently enriched across different photodermatoses. Although microbial profiling in many studies has been performed primarily at the genus level, which limits detailed interpretation at the species or strain level, it remains uncertain whether the *S. aureus* detected in different photodermatoses represents similar strains or whether its role in disease varies across clinical subtypes. Moreover, its interpretation is further complicated by the substantial variability of the skin microbiome across anatomical sites and between individuals [16]. Sebaceous, moist and dry skin sites harbor distinct microbial communities, and interindividual variation among healthy subjects may be comparable to, or even greater than, differences observed between healthy and diseased skin. Therefore, the enrichment of *S. aureus* should not be interpreted as sufficient to cause disease on its own; host factors, local barrier integrity, immune status, UV exposure, microbial strain differences, and interactions with other community members may determine whether colonization is associated with inflammation or disease progression. Addressing these issues in future work may help to clarify the involvement of the skin microbiome in photodermatoses.

## 3. Mechanism of Microbiota Dysbiosis Formation

Solar ultraviolet radiation (UVR) reaching the Earth’s surface consists primarily of UVA (315–400 nm) and a smaller proportion of UVB (280–315 nm), whereas UVC is largely absorbed by atmospheric ozone. UVA accounts for most daily terrestrial UV exposure and penetrates more deeply into the dermis, while UVB represents a smaller fraction of sunlight but has higher photon energy and is more efficient at inducing epidermal DNA photoproducts, erythema, and inflammatory responses [35]. The relative intensity of UVA and UVB varies with solar elevation, season, latitude, altitude, cloud cover, and ozone concentration, with UVB showing more pronounced midday variation than UVA [35]. These wavelength- and exposure-dependent differences may influence both host cutaneous responses and the composition or activity of skin-associated microorganisms [21,36].

### 3.1. UVR Changes the Skin Microbiome by Affecting Antimicrobial Peptides

Human skin antimicrobial peptides (AMPs) include β-defensins (HBD1, HBD2, HBD3, HBD4), LL-37, psoriasin, RNase7, and others [37,38]. These AMPs not only have bactericidal activity, but also participate in immune regulation and the activation of adaptive immunity [37,38]. They can act as chemotactic factors to recruit immune cells and promote the maturation and activation of antigen-presenting cells [37,38]. Both UVR and commensal bacteria can stimulate keratinocytes to produce AMPs [39,40,41]. For example, *S. epidermidis* and *C. acnes* stimulate skin cells to produce HBD-2, HBD-3 and LL-37, reflecting the immune crosstalk between the skin microbiota and host skin cells [36,37,42].

Dysregulation of the AMP profile has been implicated in several skin diseases, such as psoriasis and AD [43,44]. Alterations in AMP expression have also been observed in PLE patients, with skin AMPs atypically upregulated after UVR exposure [43]. Key observations include the following: (1) psoriasin is significantly upregulated and exhibits diffuse expression across the entire epidermis, rather than showing focal increases as in healthy skin; (2) HBD-2 is higher in PLE patients than in healthy individuals and is further increased in lesional skin; (3) HBD-3 exhibits no significant difference between PLE patients and healthy individuals; and (4) RNase7 and LL-37 expression levels are significantly higher in the skin of PLE patients than in healthy individuals. The atypical AMP profile of PLE patients is similar to that in psoriasis patients, but differs from that in AD patients, even though PLE patients and AD patients are both characterized by *S. aureus* colonization. It is likely that the differences in the skin microbiota between AD and PLE may contribute to their similar yet distinct AMP expression profiles [43,44].

Furthermore, a vitamin D3 analog, calcipotriol, can alleviate symptoms in PLE patients, and is believed to regulate AMP expression via the Th17 pathway. In vitro studies on keratinocytes have also shown that calcipotriol inhibits certain AMPs stimulated by UVB [45]. Given that microbes may influence the production of Th17-associated cytokines, thereby affecting AMP expression [36,46], these findings suggest a potential involvement of microbes and AMPs in PLE pathogenesis. An atypical AMP milieu could exert selective pressure on the skin microbiota, potentially promoting opportunistic species, such as *S. aureus,* while suppressing certain commensals, thus setting the stage for microbial dysbiosis in photodermatoses.

### 3.2. The Direct Effect of UVR on the Microbiome

UVR not only influences the skin microbiome indirectly through host responses, but also directly affects microorganisms. UVR induces the production of reactive oxygen species, leading to oxidative damage to nucleic acids, proteins, and lipids. Furthermore, UVR can directly damage DNA, inducing the formation of photoproducts. Collectively, UVR exerts bactericidal and bacteriostatic effects. Studies indicate that these effects are spectrum-, dose-, and strain-dependent [11]. Hence, UVR may disrupt skin microbiome composition, facilitating microbial dysbiosis [11,21,36].

Studies in healthy individuals have shown that UVR exposure is associated with changes in skin microbial composition, including an increased abundance of *Cyanobacteria* and *Fusobacteria*, as well as a decreased abundance of *Lactobacillaceae* and *Pseudomonadaceae*. Among these taxa, *Lactobacillus* spp. and *Clostridium* have been reported as relatively enriched genera in the healthy skin microbiome [21,46,47].

Notably, UVR-associated microbial shifts appear to differ between patients with photodermatoses and healthy individuals. For example, *Cyanobacteria* show a clear decrease in PLE patients following UVR exposure [11], whereas an increase has been observed in healthy individuals after UVB and UVA1 exposure [47]. Whether such differences reflect variations in UVR dose, spectrum, and exposure frequency, or differences in sampling time points, remains unclear.

### 3.3. Reduction or Attenuation of Commensal Bacteria

Another contributor to microbial dysbiosis is the reduction or attenuation of commensal bacteria that normally suppress *S. aureus* and its virulence. In diseases such as AD and AK, the reduction or decreased activity of other commensal bacteria can lead to a microbiome imbalance favoring *S. aureus* [9].

Coagulase-negative staphylococci (CoNS) isolated from healthy individuals exhibit antibacterial activity against *S. aureus*, partly through the secretion of AMPs that act together with human-derived LL-37. CoNS can also inhibit the virulence of *S. aureus* [48]. However, CoNS are deficient in AD patients [14]. Similarly, PLE patients also show decreased CoNS, suggesting a similar loss of protective staphylococci in photodermatoses characterized by *S. aureus* colonization [11]. Moreover, *Cutibacterium* and *Malassezia* are negatively correlated in abundance with *S. aureus*, and downstream analyses suggest active competition and/or antagonism [9]. *Cutibacterium acnes* can also directly inhibit *S. aureus* by producing propionic acid and competing for nutrients [20]. The *Corynebacterium* genus is significantly reduced in PLE patients [47]. Notably, *Corynebacterium striatum*, one of the most common species within this genus, can modulate the biological behavior of *S. aureus*, thereby suppressing its virulence [49].

### 3.4. Immune-Mediated Barrier Impairment Promotes Microbial Dysbiosis

One of the most important immune-mediated mechanisms involves the upregulation of type I interferons (IFN). Patients with SLE and CLE exhibit increased type I IFN signaling in the blood and skin, and UVB exposure can further amplify IFN-related responses in keratinocytes [50,51]. Increased type I interferons, particularly IFN-α, regulate gene expression in epithelial cells such as keratinocytes. This leads to decreased expression of skin-barrier-related proteins and increased expression of adhesion-related genes: *FLG*, *FLG2*, and *LOR* are downregulated, impairing the cornified envelope; *DSG1* is reduced, weakening cell–cell adhesion; and *FN1*, *ITGA5*, and *ITGB1* are upregulated, facilitating *S. aureus* adherence [10]. These changes may promote *S. aureus* colonization. In addition, downregulated expression of filaggrin leads to a reduction in its decomposition product, natural moisturizing factor (NMF). The decreased level of NMF results in an increased skin pH, under which *S. aureus* can proliferate rapidly [10,52,53].

**Figure 2:**
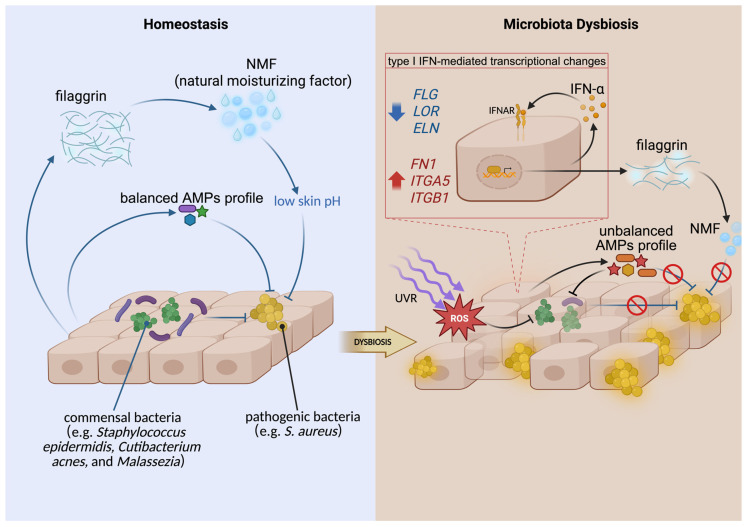
Formation mechanism of dysbiotic microbiota. Black arrows indicate direction of effect, production, secretion, or transition; blue T-bars indicate inhibitory effects; red prohibition symbols indicate inhibition or blockade. Created in BioRender. Su, F. (2026) https://BioRender.com/Iryieej (accessed on 4 May 2026).

Figure 2 summarizes the potential mechanisms underlying microbial dysbiosis in photodermatoses. Atypical AMP profiles and UVR may exert selective pressure on the skin microbiome, contributing to dysbiosis. Reduction in commensal bacteria can weaken the inhibition of *S. aureus*, which may further disrupt the skin barrier and amplify microbial imbalance. Microbes can also produce natural AMPs and stimulate keratinocytes to generate AMPs, which in turn shape the microbial community [47]. These reciprocal interactions suggest a bidirectional process, but the sequential dynamic of microbes and AMPs in photodermatoses remains incompletely understood.

Studies examining microbiome changes after UVR exposure in healthy individuals and photodermatoses patients have highlighted several outstanding questions: (1) UVR-induced microbial changes appear to differ between healthy individuals and patients, but the roles of the UVR spectrum, intensity, or sampling time are unclear; (2) microbial diversity decreases in photodermatoses patients after UVR exposure, whereas corresponding data in healthy individuals are limited; and (3) the current studies generally involve short-term sampling, and the long-term effects of UVR on the skin microbiome remain poorly characterized.

## 4. Dysbiotic Microbiome in the Occurrence of Photodermatoses

### 4.1. Inflammatory Responses Triggered by Microbial Signals

Pathogen-associated molecular patterns (PAMPs) and commensal-associated molecular patterns (CAMPs) are conserved microbial molecules, including lipopolysaccharide (LPS), lipoteichoic acid (LTA), and other substances. These molecules can be recognized by pattern recognition receptors (PRRs), such as Toll-like receptors (TLRs). Recognition of these microbial signals can activate nuclear transcription factor pathways (NF-κB, AP-1, and IRF), leading to an immune response.

In PLE, PAMPs and CAMPs may contribute to the initiation of inflammation [45]. UVR can damage DNA, proteins, and lipids within the skin microbiota, promoting the release of microbial signals that interact with PRRs on host cells. This interaction can induce AMP production and inflammatory activation. UVR-induced barrier disruption may further facilitate microbial infiltration, exacerbating inflammation triggered by microbial antigens and signals [54]. Additionally, genetic studies on PRR polymorphisms may also provide evidence consistent with this hypothesis: differences in PRRs (NOD-2 and TLR-5) have been observed between PLE patients and healthy individuals [55].

That microbial signals contribute to the early phase of PLE pathogenesis may explain why a specific photoantigen has not been identified. Unlike a type IV hypersensitivity reaction to a defined photosensitizer involving monoclonal T-cell infiltration, immune responses elicited by multiple microbial antigens and PAMPs/CAMPs are unlikely to show clonal restriction [45].

### 4.2. Microorganisms Express Virulence Factors to Damage the Skin

*S. aureus* secretes products such as hemolysins, phenol-soluble modulins, and serine proteases. These secreted products stimulate keratinocytes to express proinflammatory cytokines, including IL-6, IL-8, and TNF-α, leading to the recruitment of neutrophils, monocytes, macrophages, and other immune cells. This process has been proposed to contribute to inflammatory responses associated with AK and SCC [56]. *S. aureus* secretes proteases that damage the skin barrier [57], thereby promoting bacterial penetration into the skin and leading to the generation of microbial antigens. This process induces the production of *S. aureus*-specific TRM cells and triggers inflammation. Under certain conditions, such as psoriasis and AD, the massive accumulation of commensal bacteria-specific T cells may contribute to disease exacerbation [54]. *S*. *aureus* secretes superantigens that bind to *HLA-DR* molecules, resulting in the activation of T cells, as well as the subsequent release of cytokines and inflammatory mediators [57]. *Malassezia* exhibits proinflammatory properties under UVR exposure. Although no clear imbalance in *Malassezia* colonization has been observed in photodermatoses so far, *Malassezia*-associated dermatoses—such as rosacea and seborrheic dermatitis—commonly occur in UV-exposed areas. Therefore, the relationship between *Malassezia* and photodermatoses requires further investigation.

### 4.3. Loss of Commensal Bacteria Benefits

The reduction in beneficial commensal microorganisms not only promotes the overgrowth of pathogens such as *S. aureus* but also weakens the protective function of the skin barrier [14,23,42,58].

*Cutibacterium acnes* plays an essential role in maintaining skin homeostasis by regulating lipid metabolism, producing propionic acid, modulating immune responses, and alleviating oxidative stress. However, its abundance is decreased in AK and SCC lesions, which may contribute to oxidative stress and inflammation, thereby promoting disease progression [9]. *Malassezia* can synthesize a UV-absorbing indole alkaloid named pityrosporum [59]. Its abundance is also found to be decreased in AK and SCC lesions. *Lactobacillaceae* are also involved in maintaining skin homeostasis and mediating anti-inflammatory effects. Nevertheless, their relative abundance decreases following UVR exposure [21], which may further contribute to the development and progression of photodermatoses.

### 4.4. UVR and Microbes Contribute to the Activation of Skin TRM

Tissue-resident memory T (TRM) cells constitute a major T-cell population in human skin, accounting for approximately half of cutaneous T cells [60]. Although they provide local immune protection, sustained antigenic or inflammatory stimulation can render them pathogenic, contributing to chronic inflammation and relapse [61,62]. In psoriasis, AD, CLE [63,64,65,66,67], and immunologically mediated photodermatoses such as PLE and CAD [68,69], TRM cells are regarded as key mediators linking environmental triggers to persistent skin inflammation.

Microbial signals contribute to TRM recruitment and maintenance [61,70,71]. IL-23 dependently supports TRM17 cells after *Candida albicans* challenge [72], and CCL20-mediated recruitment of CCR6^+^ TRM17 cells by commensal fungi illustrates this interaction [70]. In psoriasis models, streptococcal-driven Th17 responses can give rise to skin TRM cells and aggravate inflammation [73]. Barrier disruption facilitates this process. Inflammatory damage permits microbial antigens to access deeper skin compartments, promoting the expansion of commensal-specific TRM populations [3]. Such feedback between barrier impairment, microbial exposure, and TRM activation has been described in psoriasis and AD [54,74] and has also been proposed in PLE, where UVR may activate commensal-specific TRM and contribute to recurrent lesions [54].

**Figure 3:**
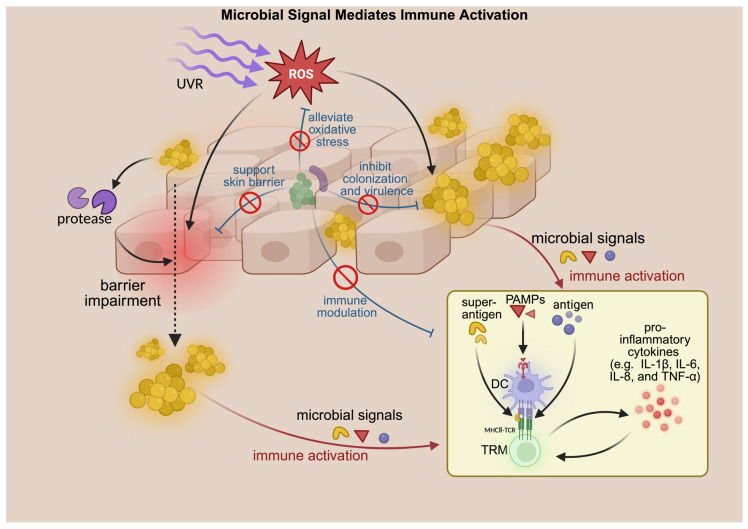
Potential contribution of microbial dysbiosis to the occurrence and progression of photodermatoses. Black arrows indicate direction of effect, production, secretion, transition, or activation; red arrows indicate microbial-signal-mediated immune activation; blue T-bars indicate inhibitory effects; black dashed arrows indicate penetration across an impaired skin barrier; red prohibition symbols indicate inhibition or blockade. Created in BioRender. Su, F. (2026) https://BioRender.com/kvosief (accessed on 4 May 2026).

UVR and microbial stimuli likely cooperate in sustaining TRM responses (Figure 3). UVR-induced cytokines (e.g., IL-1β, IL-6, TGF-β) [75,76], barrier damage [77], and enhanced microbial antigen exposure together promote TRM activation, while TRM-driven inflammation may further reshape the local microbiome, perpetuating disease activity.

Taken together, microbial signals and virulence factors may be involved in early inflammatory responses in photodermatoses, while barrier impairment, microbial imbalance, and TRM activation appear more closely related to disease persistence and recurrence (Figure 2 and Figure 3). Supporting evidence from PLE shows that reducing the skin microbial load can attenuate UV-induced skin responses, suggesting a potential role of microbes in disease development [11,13]. However, several limitations remain. Much of the evidence on *Staphylococcus aureus* is derived from atopic dermatitis, and its relevance to other photodermatoses requires further validation. In addition, the current data do not clarify whether microbiome changes precede disease onset, reflect alterations in the skin microenvironment, or contribute to disease progression. Future studies integrating microbiome and host immune analyses may help clarify these relationships.

## 5. New Therapeutic Strategies Targeting Microorganisms

Photodermatoses with microbiome dysbiosis may benefit from therapies that either eliminate pathogenic microbes or bolster beneficial ones. Treatments targeting *S. aureus* have been proven to alleviate symptoms in AD patients, and skin disinfection has been reported to partially reverse the cytokine dysregulation in PLE patients [13]. Considering the crucial role of the microbiome and the confirmed microbial dysbiosis in patients with photodermatoses, it is reasonable to hypothesize that microbes may serve as a potential target for early intervention.

Based on the mechanism by which the microbiome may contribute to photodermatoses, potential therapeutic strategies can be summarized as follows: (a) microbe-targeted therapies, aimed at reducing or eliminating pathogenic bacteria; and (b) microbiome-enhancing strategies, aimed at restoring healthy microbial balance (Table 2).

### 5.1. Microbe-Targeted Therapies

This category encompasses interventions designed to reduce the burden of pathogenic microorganisms, particularly *S. aureus*, thereby attenuating microbial-driven inflammation and barrier disruption.

#### 5.1.1. Antibiotics

Antibiotics are a traditional strategy for targeting pathogenic bacteria, and have been used in several dermatological conditions associated with microbial dysbiosis. In these cases, antibiotics aim to reduce pathogen colonization burden, rather than restore the entire skin microbiome by themselves. Several examples are summarized in Table 2.

However, antibiotics have several limitations. First, their clinical benefits do not always parallel the reductions in *S. aureus* colonization; some interventions can reduce *S. aureus* colonization without providing additional clinical improvement, and recolonization may occur after treatment [78]. Second, bacterial resistance remains a concern, and repeated or prolonged antibiotic exposure may further this effect in resistant organisms [15,79]. Indeed, methicillin-resistant *S. aureus* (MRSA) and even mupirocin-resistant strains have been reported in chronic skin disease [79]. In addition, broad or repeated antibiotic use may adversely affect beneficial skin commensals [15].

To minimize adverse effects on beneficial bacteria, intranasal antibiotic application has been proposed as an alternative approach. The nasal cavity is the main reservoir for *S. aureus*, with persistent colonization observed in approximately 20% of healthy individuals. One sequencing study suggests that cutaneous *S. aureus* may originate from the nares [9]. Notably, the nasal carriage of *S. aureus* is associated with an increased risk of infection and secondary skin colonization [9]. Intranasal antibiotic therapy has shown promising results in microbe-associated dermatoses such as AD [19], where it effectively reduces *S. aureus* colonization on the skin of AD patients [15]. Given that certain types of photodermatoses also exhibit abnormal *S. aureus* colonization, the intranasal application of antibiotics may represent a potential therapeutic option for these conditions. However, this strategy remains speculative, requiring clinical studies. In all cases, antibiotic stewardship is crucial—using the narrowest spectrum and shortest duration needed—to avoid fostering resistant microbes and to preserve the resident microbiome.

**Table 2:** Antibiotics and decolonization strategies used in dermatological conditions associated with microbial dysbiosis.

Antibiotic	Application	Disease/Context	Microbiome Dysbiosis/Infection	Reference
Mupirocin	Topical	Atopic eczema (atopic dermatitis)	*S. aureus*	(2019) George et al. [78]
Mupirocin	Intranasal, plus bleach bath	Atopic eczema (atopic dermatitis)	*S. aureus*	(2009) Huang et al. [80]
Cefuroxime	Oral	Atopic eczema (atopic dermatitis)	*S. aureus*	(2019) Van et al. [81]
Minocycline	Oral	Acne vulgaris	*C. acnes*	(2019) Gold et al. [82] (2020) Thompson et al. [83]
Doxycycline	Oral	Acne vulgaris	*C. acnes*	(2021) Eichenfield et al. [84]
Clindamycin	Topical	Acne vulgaris	*C. acnes*	(2021) Eichenfield et al. [84]
Tetracyclines, clindamycin, and rifampicin	Oral	Hidradenitis suppurativa	Prevotella, Porphyromonas, Fusobacterium	(2020) Van Straalen et al. [85]
Mupirocin	Topical	Cutaneous lupus erythematosus	*Staphylococcus* spp.	(2025) Abernathy-Close et al. [86]

#### 5.1.2. Phototherapy and Photodynamic Therapy

Phototherapy exerts anti-inflammatory effects and reduces the colonization of pathogenic bacteria, and is thereby applied in the treatment of certain dermatoses. For instance, blue light, red light, and narrowband UVB (NB-UVB) are applied in the treatment of mild-to-moderate acne vulgaris [87,88]; and psoralen plus UVA (PUVA), UVA1, and NB-UVB are applied in the treatment of AD [89].

Phototherapy is generally regarded as a contraindication for photodermatoses. Nevertheless, during the remission phase of certain photodermatoses, low-dose phototherapy can be used preventively after careful clinical evaluation [90]. This approach is based on the so-called “hardening phenomenon” in photodermatoses. The potential mechanisms driving the preventive effect include increased melanin pigmentation, thickening of the stratum corneum, the depletion of putative photoantigens, the elevation of serum vitamin D3 levels, and the modulation of immune response. Multiple studies have demonstrated that NB-UVB or PUVA phototherapy exhibits favorable clinical efficacy in patients with PLE [91,92]. Additionally, phototherapy serves as a second-line therapy for CAD patients and has potential preventive applications [90]. Nevertheless, the mechanisms underlying the impact of phototherapy on microorganisms remain to be further elucidated. For instance, phototherapy in AD patients reduces the abundance of *S. aureus* on the skin and alleviates disease symptoms. In contrast, patients with PLE and CAD exhibit increased *Staphylococcus* abundance and decreased microbial diversity following UVR exposure, as noted earlier. These discrepancies may be associated with factors such as the intensity and spectrum of the ultraviolet light employed, as well as the strain of *S. aureus* involved.

Photodynamic therapy (PDT) relies on reactive oxygen species (ROSs)—generated by photosensitizers upon light excitation in an oxygen-rich environment—to exert a cytotoxic effect on target cells. Aminolaevulinic acid–photodynamic therapy (ALA-PDT) has long been an effective approach for treating moderate-to-severe acne vulgaris [93]. In addition to ablating affected sebaceous cells, ALA-PDT can also inhibit *C. acnes* and improve skin microbial diversity in acne patients.

In terms of photodermatoses, PDT can be used for conditions such as AK [94], primarily by eliminating abnormally proliferating keratinocytes. However, PDT remains contraindicated in most photodermatoses. Interestingly, PDT has been shown to inactivate *S. aureus* and its virulence factors [95], suggesting that localized PDT within the nasal cavity may help reduce nasal *S. aureus* carriage and consequently decrease cutaneous colonization.

#### 5.1.3. Disinfectants or Bleach Baths

Disinfectants or bleach baths are commonly used in the management of AD. Their mechanism of action is thought to involve reducing the burden of *S. aureus* while permitting the expansion of commensal bacterial populations, thereby increasing microbial diversity [19]. Given that the dysregulation of *S. aureus* has been confirmed in photodermatoses, similar decolonization approaches may hold therapeutic potential in these conditions. Additionally, bleach baths can attenuate cutaneous inflammation by suppressing the MAPK- and NF-κB-signaling pathways [15]. The combined use of bleach baths with intranasal mupirocin has been demonstrated to further decrease *S. aureus* colonization and to significantly improve the clinical outcomes of AD patients [15,19].

#### 5.1.4. Phage Therapy

Compared with traditional antibiotic therapy, phage therapy offers distinct advantages in targeting drug-resistant bacteria, and recent advances in bioengineering technologies have further expanded its clinical potential [96]. The strain-specific property of phages offers significant advantages, one notable example is SaGU1, a phage strain that can specifically target *S. aureus* while sparing *S. epidermidis* [97].

Phage therapy has demonstrated efficacy in various infectious murine models, effectively targeting several species of Gram-negative bacteria, including *Acinetobacter baumannii*, *Pseudomonas aeruginosa*, and *Vibrio vulnificus*. Additionally, phage administration can rescue mice challenged with a lethal dose of *S. aureus*, confirming the therapeutic potential of this approach [15].

Combining phage therapy with microbiota-based strategies may enhance the therapeutic effect. As noted earlier, the combined use of SaGU1 and *S. epidermidis* in treating AD patients has shown promising therapeutic results, and this approach also provides a new strategy for tackling phage-resistant bacterial strains.

However, phage therapy still faces important limitations. For instance, oral administration may disrupt the intestinal barrier and exacerbate inflammatory bowel diseases such as Crohn’s disease [98]. Potential solutions include the co-administration of oral phages with probiotic formulations, the use of localized phage applications, or the development of genetically engineered phages to improve safety and specificity.

### 5.2. Microbiome-Enhancing Strategies

This category focuses on harnessing the photoprotective, anti-inflammatory, and antioxidant properties of beneficial microorganisms to restore microbial balance and promote skin homeostasis.

#### 5.2.1. Microbiota Therapy

Microbiota therapy can be categorized into probiotics, prebiotics, postbiotics, and microbiota transplantation. Probiotics, for instance, can compete with pathogenic bacteria, secrete bioactive metabolites, lower the skin pH, and form physical barriers or biofilms that inhibit pathogen adhesion. Both probiotics and prebiotics exhibit photoprotective properties through their antioxidant, anti-inflammatory, anti-aging, anti-carcinogenic, and melanin-promoting effects [59].

Microbiota therapy has been widely demonstrated to improve dermatoses associated with microbial dysbiosis [59]. The following bacterial strains have been shown to exert inhibitory effects on *S. aureus* and may offer potential therapeutic benefits in alleviating photodermatoses (Table 3):

• *Staphylococcus epidermidis*

*S. epidermidis* can alleviate photodermatoses by inhibiting *S. aureus* colonization, suppressing inflammation, and promoting tissue repair. It has been demonstrated to reduce *S. aureus* colonization and virulence in AD patients. The mechanism may involve three major pathways: (1) the secretion of the serine protease Esp, which degrades adhesion-related proteins required for *S. aureus* colonization; (2) the production of the α-soluble mediator, free fatty acids, and the synergistic secretion of LL-37 with keratinocytes, which inhibits *S. aureus* [99]; and (3) the secretion of autoinducing peptides, which inhibit the accessory gene-regulatory (agr) quorum-sensing system, thereby inhibiting the virulence of *S. aureus* [48].

In addition, *S. epidermidis* exhibits anti-inflammatory effects by promoting the differentiation of naive T cells into FOXP3^+^ Treg cells expressing CTLA4 and ICOS14, thereby mediating immune tolerance. In adult mice colonized with *S. epidermidis*, Foxp3^+^ Treg cells persist around hair follicles and suppress abnormal inflammatory responses [99]. Moreover, short-chain fatty acids (e.g., succinic acid) produced by *S. epidermidis* have been demonstrated to reduce local inflammation [59]. Non-invasive *S. epidermidis* also drives the establishment of specific CD8^+^ TRM cells via non-classical MHC-Ib H2-M3 peptide presentation; these TRM cells are essential for tissue repair and wound healing [100].

• *Corynebacterium striatum*

Matthew et al. proposed a therapeutic strategy that modifies *S. aureus* behavior rather than eliminating it. *Corynebacterium striatum* interacts with *S. aureus* and reduces its virulence, shifting *S. aureus* from a pathogenic to a commensal state through phenotypic transformation [49]. Consistently, Brown et al. found that *S. epidermidis* may either maintain or disrupt the skin barrier, depending on its microenvironment and strain type [101], suggesting the behavioral plasticity and diversity of the skin microbiota.

• *Cutibacterium acnes*

*Cutibacterium acnes* (*C. acnes*) plays a key role in maintaining skin homeostasis by regulating lipid metabolism, competing within the follicular microenvironment, modulating immune responses, and mitigating oxidative stress. Notably, its abundance is negatively correlated with *S. aureus* colonization in AD patients. The free radical oxygenase RoxP produced by *C. acnes* exhibits strong antioxidant activity, protecting skin cells from UV-induced damage by preventing free radical formation [102]. Recent research indicates that reduced diversity among the *C*. *acnes* subspecies—rather than its proliferation—is related to acne development [32]. This finding provides a basis for therapeutic use of *C. acnes* while avoiding acne-related side effects.

• *Rhodospirillum roseum*

The effects of *Rhodospirillum roseum* (*R. roseum*) appear strain-specific: isolates derived from AD patients exacerbate inflammation in murine models, whereas those from healthy individuals reduce *S. aureus* colonization in AD-like mice and AD patients [103,104].

• *Malassezia*

*Malassezia* can synthesize an UV-absorbing indole alkaloid named pityrosporum [59]. This finding indicates that *Malassezia* or its bioactive metabolite could be applied in microbiota-based photoprotection therapies.

• *Lactobacillus reuteri*

Topical application of active *Lactobacillus reuteri* (*L. reuteri*) reduces UVB-induced inflammation and exhibits antibacterial activity against *S. aureus*. It upregulates the expression of the aquaporin 3 (*AQP3*) gene, and its lysate increases laminin A/B levels, collectively indicating beneficial effects on the skin barrier [105].

• *Lactobacillus johnsonii*

*Lactobacillus johnsonii* maintains the population and function of Langerhans cells after UV exposure, thereby preserving skin immunity, reducing skin inflammation, and alleviating photoaging [99].

• *Lactobacillus rhamnosus*

In vitro, *Lactobacillus rhamnosus* CNCM I-2116 (ST11) inhibits substance P-induced skin inflammation and accelerates skin barrier recovery [99]. Additionally, its lysate further contributes to skin barrier reconstruction [106].

• *Lactobacillus acidophilus*

Oral administration of a nutritional supplement containing lycopene, β-carotene, and *Lactobacillus acidophilus* (*L. acidophilus*) can alleviate UVA-induced PLE [45]. Inactivated *L. acidophilus* also scavenges ROSs associated with UVB-induced oxidative stress [107].

• Lactic acid bacterium isolate XJC60

The *lactic acid bacterium* isolate XJC60 exhibits UV-protective effects by producing nicotinamide, a UV-protective metabolite. Additionally, the strain displays a high level of free radical scavenging capacity, reducing ROS levels by 70% and stabilizing the mitochondrial membrane potential [108].

• *Bifidobacterium*

The lysate of *Bifidobacterium longum* (*B. longum*) reduces various inflammation-related parameters, including vasodilation, edema, mast cell degranulation, and TNF-α release, indicating its potential to decrease skin sensitivity and enhance resistance to physical stress [99]. Similarly, oral administration of *Bifidobacterium breve* (*B. breve*) prevents UV-induced transepidermal water loss (TEWL) and reduces cutaneous hydrogen peroxide levels, protein oxidation, and xanthine oxidase activity [59].

• *Cyanobacteria*

*Cyanobacteria* synthesize UV-absorbing compounds such as mycosporine-like amino acids (MAAs) and sphingosine. Notably, MAAs dissipate the absorbed UV radiation as heat without generating ROSs. Additionally, *Cyanobacteria* can synthesize vitamin C, vitamin E, carotenoids, and reduced glutathione to alleviate the oxidative stress induced by UVA. Furthermore, these organisms synthesize enzymes that combat oxidative stress, including superoxide dismutase and catalase [21]. Collectively, these features highlight the potential of *Cyanobacteria* and their derivatives in the treatment of photodermatoses.

At present, microbiota therapy still has its limitations. Key challenges include the thermal instability of probiotics, opportunistic pathogenicity, and the potential transfer of antibiotic resistance genes. Rigorous safety evaluations are essential. In addition, genetic engineering modifications and the utilization of microbial metabolites or postbiotics are viable strategies to minimize these risks [99].

**Table 3:** Bacterial strains that may be beneficial for alleviating photodermatoses.

Bacterial Strains	Mechanism	Reference
*Staphylococcus epidermidis*	serine protease Esp: inhibits *S. aureus* colonization	Patra V. et al. [59] Gueniche A. et al. [99] Linehan JL et al. [100]
	α-soluble mediator, free fatty acids, and LL-37: inhibits *S. aureus*	
	promotes the differentiation of naive T cells into FOXP3^+^ Treg cells	
	succinic acid: reduces inflammation	
	enables the establishment of specific CD8^+^ TRM: tissue repair and wound healing	
*Corynebacterium striatum*	reduces the virulence of *S. aureus*	Ramsey MM et al. [49] Brown MM et al. [101]
*Cutibacterium acnes*	lipid metabolism regulation, follicular microenvironment competition, immune regulation, and alleviating oxidant stress	Andersson T et al. [102]
	RoxP: UVR protection	
*Rhodospirillum roseum*	reduces *S. aureus* colonization	Ia M et al. [104]
*Malassezia*	pityrosporum: absorbs UVR	Patra V et al. [59]
*Lactobacillus reuteri*	reduces inflammation induced by UVB	Khmaladze I et al. [105]
	antibacterial activity against *S. aureus*	
	upregulates the expression of AQP3 gene	
	elevates the levels of laminin A/B	
*Lactobacillus johnsonii*	maintains the number and function of Langerhans cells after UVR	Gueniche A et al. [99]
*Lactobacillus rhamnosus*	inhibits skin inflammation induced by substance P	Gueniche A et al. [99] Yo J et al. [106]
	accelerates the recovery of the skin barrier	
*Lactobacillus acidophilus*	scavenges reactive oxygen species (ROSs) generated by UVB-induced oxidative stress.	Patra V et al. [45]
Lactic acid bacterium isolate XJC60	free radical scavenging capacity	Chen H et al. [108]
*Bifidobacterium*	reduces inflammation	Patra V et al. [59] Gueniche A et al. [99]
	prevents transepidermal water loss induced by UVR	
*Cyanobacteria*	mycosporine-like amino acids: absorbs radiation	Burns EM et al. [21] Gueniche A et al. [99]
	synthesizes vitamin C, vitamin E, carotenoids, and reduces glutathione	

#### 5.2.2. Next-Generation Sunscreen

Sunscreen is essential for the management and treatment of photodermatoses; however, it exerts a dual influence on the skin microbiome [46,109,110]. On one hand, sunscreen provides UV protection and reduces UVR-induced microbial perturbations [110]. On the other hand, multiple sunscreen ingredients may disturb skin microbial homeostasis. For instance, zinc oxide nanoparticles, widely used in inorganic UV filters, can increase cell membrane permeability at high concentrations, exerting bacteriostatic or bactericidal effects [46,109]. Preservatives and antimicrobial additives incorporated into sunscreen formulations may further exert broad microbial suppression. Additionally, sunscreens may modify local skin pH, which in turn influences microbial colonization, competition, and metabolic activity [109].

Theoretically, next-generation sunscreens should possess the following properties: (1) fewer microbial-perturbing ingredients; (2) the incorporation of prebiotics and postbiotics, which have been explored to help maintain the diversity of the skin microbiome [111,112]; and (3) a physiologically acidic pH. A physiologically acidic pH of the skin provides twofold benefits: it inhibits the colonization and pathogenicity of *S. aureus*, and simultaneously supports the survival of commensal bacteria and facilitates skin barrier repair [113].

Collectively, maintaining microbial diversity, supporting commensal–host interactions, and leveraging microbiota-derived bioactive molecules represent promising development directions for next-generation skincare products [46,109].

## 6. Discussion

An increasing number of studies have revealed the close connection between dermatoses and microbiome dysbiosis; thus, the role of microbial imbalance in photodermatoses has gained growing attention. This review summarizes the current findings on microbial alterations in photodermatoses, their possible mechanisms of formation, and their pathogenic implications. On this basis, we propose a conceptual framework integrating UV radiation, microbiome dysbiosis, host immune responses, and TRM cells as a central immunological hub. Within this framework, acute lesion development may be driven by inflammation initiated by microbial signals and toxins, whereas barrier impairment, persistent dysbiosis, and sustained activation of TRM cells may together contribute to disease chronicity.

However, this framework should be interpreted with caution. Most available studies are observational or cross-sectional, and therefore demonstrate associations rather than causation. Disease-associated changes in the skin microenvironment may reshape microbial colonization, and substantial microbiome variability across anatomical sites and between individuals may confound disease-associated microbial signatures. Although interventional studies in AD and experimental PLE models suggest that microbiome manipulation can influence skin disease severity or UV-induced immune responses, comparable clinical evidence directly demonstrating that microbiome alteration changes disease outcomes in photodermatoses remains lacking.

From a clinical perspective, these observations suggest that photodermatoses may benefit from an integrated assessment of the skin microbiome, immune status, and clinical phenotype. Although still exploratory, insights into microbiota–immune interactions and TRM biology point toward potential therapeutic strategies. Modulation of the skin microbiome may be considered as an adjunctive approach in early or mild disease, while in more chronic or severe conditions, immunosuppressive treatment may induce remission, followed by maintenance strategies aimed at restoring barrier function, rebalancing the microbiota, and limiting pathogenic TRM activity to reduce relapse.

Despite increasing correlative evidence supporting a UV–microbiota–host immunity axis, mechanistic understanding remains limited. Many current models are based on indirect observations, and direct experimental validation is still lacking. Future studies would benefit from more standardized and integrative approaches, including longitudinal and paired sampling designs to capture dynamic microbial and immune changes after UVR exposure, improved taxonomic resolution to distinguish pathogenic and protective microbial behaviors, and multi-omics analyses integrating microbiome data with host immune and barrier-related pathways. In addition, functional studies using appropriate experimental models, as well as carefully designed clinical investigations of microbiota- or TRM-targeted interventions, will be important for translating these concepts into clinical practice.

Overall, advancing the field will require coordinated efforts that integrate photobiology, barrier biology, microbial ecology, and host immunity. Such an approach may ultimately provide a more comprehensive understanding of photodermatoses’ pathogenesis and inform the development of more precise diagnostic and therapeutic strategies.

## Figures and Tables

**Table 1:** Reported associations between microbiome dysbiosis or alterations and several photosensitive and photo-related skin diseases.

Diseases	Microbiome Dysbiosis/Alterations	Analysis Method	Subjects	Reference
Polymorphous light eruption	↑*Staphylococcus aureus* ↓*Staphylococcus epidermidis* ↓ *Staphylococcus hominis* ↓ *Cutibacterium acnes* ↓ *Corynebacterium* ↓ *Cyanobacteria*	16S rRNA sequencing	11 PLE patients and 11 healthy controls	(2025) Amar Y. et al. [11]
Hydroa vacciniforme	EBV	Real-time polymerase chain reaction	30 patients and 24 healthy individuals	(2020) Miyake T. et al. [28]
Chronic actinic dermatitis	↑ *Staphylococcus* ↓ *Cutibacterium*	16S rRNA sequencing	15 CAD patients and 14 matched controls	(2025) Tu Y. et al. [12]
Actinic keratosis and cutaneous squamous cell carcinoma	↑ *Staphylococcus aureus* ↓ *Cutibacterium* ↓ *Malassezia*	16S/18S rRNA sequencing	13 SCC-prone immunocompetent men	(2018) Wood D. L. A. et al. [9]
Lupus erythematosus	↑ *Staphylococcus aureus*	16S rRNA sequencing	69 SLE patients, 49 healthy controls, and 20 dermatomyositis (DM) patients	(2020) Huang C. C. et al. [25]
Rosacea	↑ *Demodex folliculorum* ↑ *Bacillus oleronius* ↑ *Staphylococcus epidermidis* ↓ *Cutibacterium acnes*	Reflectance confocal microscopy counting; 16S rRNA clone libraries; 16S rRNA sequencing	60 rosacea patients and 40 controls; 30 rosacea patients and 17 healthy controls; 17 rosacea patients and 27 healthy controls	(2017) Erdemir A. T. et al. [29] (2014) Murillo N. et al. [30] (2023) Xiong J.X. et al. [31]
Acne vulgaris	↑ *Cutibacterium acnes IA1*	Multi- and single-locus sequence typing	24 acne patients and 12 healthy controls	(2018) Dagnelie MA. et al. [32]
Psoriasis	↑ *Staphylococcus aureus* ↓ *Staphylococcus epidermidis* ↓ *Cutibacterium acnes*	16S rRNA sequencing	28 psoriasis patients and 26 healthy controls	(2018) Chang HW. et al. [33]
Atopic eczema (atopic dermatitis)	↑ *Staphylococcus aureus*	16S rRNA sequencing	95 AD patients and 77 healthy controls	(2024) Yang XP. et al. [34]

Note: These studies show associations and do not determine whether dysbiosis is a cause or consequence of disease. ↑ indicates increased abundance or colonization; ↓ indicates decreased abundance or colonization.

## Data Availability

No new data were created or analyzed in this study. Data sharing is not applicable to this article.
